# Cumulative Residual Entropy of the Residual Lifetime of a Mixed System at the System Level

**DOI:** 10.3390/e25071033

**Published:** 2023-07-09

**Authors:** Mohamed Kayid, Mashael A. Alshehri

**Affiliations:** 1 Department of Statistics and Operations Research, College of Science, King Saud University, Riyadh 11451, Saudi Arabia; drkayid@ksu.edu.sa; 2 Department of Quantitative Analysis, College of Business Administration, King Saud University, Riyadh 11362, Saudi Arabia

**Keywords:** coherent system, cumulative residual entropy, Shannon entropy, system signature

## Abstract

Recently, there has been growing interest in alternative measures of uncertainty, including cumulative residual entropy. In this paper, we consider a mixed system consisting of *n* components, assuming that all components are operational at time *t*. By utilizing the system signature, we are able to compute the cumulative residual entropy of a mixed system’s remaining lifetime. This metric serves as a valuable tool for evaluating the predictability of a system’s lifetime. We study several results related to the cumulative residual entropy of mixed systems, including expressions, limits, and order properties. These results shed light on the behavior of the measure and provide insights into the predictability of mixed systems. In addition, we propose a criterion for selecting a preferred system based on the relative residual cumulative entropy. This criterion is closely related to the parallel system and provides a practical way to choose the best system configuration. Overall, the present study of cumulative residual entropy and the proposed selection criterion provide valuable insights into the predictability of mixed systems and can be applied in various fields.

## 1. Introduction

Exploring distribution functions with limited information involves a host of compelling activities, such as poverty assessment, model selection, portfolio analysis, hypothesis testing, and estimation. The entropy measure of a probability distribution has a diverse range of applications across multiple fields including statistics, physics, economics, information sciences, and communication theory. The origin of this measure can be traced back to Shannon’s extensive article [1]. If *X* is an absolutely continuous non-negative random variable with the probability density function (pdf) f(x), the Shannon differential entropy can be defined as H(X)=−E[logf(X)] (provided that the expectation exists). Due to its versatility and practicality, this measure has become widely used in many areas of research.

Despite the many advantages of differential entropy, Rao et al. [2] proposed an alternative measure called cumulative residual entropy (CRE). This measure is obtained by replacing the pdf f(x) with the survival function S(x)=P(X>x) and is defined as follows:

(1) $$E\left(X\right)=-\int_{0}^{\infty }S\left(x\right)\log S\left(x\right)dx,$$

provided that the integral exists. Rao et al. [2] noted that for the finiteness of CRE, it is necessary to have ${E|X|}^{p}<\infty$ for some p>N, where *N* is a natural number. The CRE is particularly well-suited for describing the dispersion of information in problems related to the aging properties of reliability theory. This measure has been used in various studies, including those by Asadi and Zohrevand [3], Baratpour [4], Baratpour and Habibi Rad [5], Navarro et al. [6], Rao [7] and Toomaj et al. [8], among others. As an example, Asadi and Zohrevand [3] demonstrated that the CRE is the expected value of the mean residual life (MRL) function m(x)=E(X−x|X>x), which can be expressed as E(m(X))=E(X).

For engineers, it is crucial to perform and quantify uncertainty in the lifetime of a system. The reason for preferring systems with lower uncertainty and longer lifetimes is that reliability tends to decrease as uncertainty increases. This concept has been studied extensively, as demonstrated by Ebrahimi and Pellery [9]. In situations where operators have some knowledge of the system’s current age, measuring the uncertainty of the system’s residual lifetime can be of interest. In such cases, the dynamic cumulative residual entropy (DCRE) is a more appropriate measure than E(X), where *X* denotes the lifetime of a new system. The DCRE is defined as follows (see Asadi and Zohrevand [3]):

E(Xt)=−∫0∞St(x)logSt(x)dx(2)=−∫0∞S(x+t)S(t)logS(x+t)S(t)dx,(3)=−∫01ψ(u)ft(St−1(u))du,

where

$$f_{t}\left(x\right)=\frac{f(x+t)}{S(t)},$$

is the pdf of $X_{t}=\left[X-t|X>t\right],$ and $S_{t}^{-1}\left(u\right)=\inf\left\{x;S_{t}\left(x\right)\geq u\right\}$ is the quantile function of $S_{t}\left(x\right)=\frac{S(x+t)}{S(t)},\;x,t>0.$ Here, we use the function ψ(u)=−ulogu,0<u<1, to define the DCRE. To apply this concept, we consider a mixed system comprising *n* components, all of which are alive at a given time *t*.

Many authors have shown a keen interest in exploring the information properties of mixed systems, as evidenced by their studies on system signatures. For instance, Toomaj and Doostparast [10,11] derived an expression for the entropy of mixed systems and established bounds for the entropy of the system’s lifetime. They also provided formulas for the Kullback–Leibler discrimination information of mixed systems and component lifetimes. Asadi et al. [12] introduced the Jensen–Shannon (JS) information criteria, a scalar function of the signature that ranks mixed systems based on their designs. They demonstrated that the JS information is always non-negative and that *r*-out-of-*n* systems attain their minimum. Most recently, Toomaj [13] and Toomaj et al. [8] delved into stochastic comparisons of R’enyi entropy and cumulative residual entropy of mixed systems, respectively, demonstrating that both systems yield similar signatures. Exciting recent research has delved into the study of coherent systems comprising *n* components, where all components are alive at time *t*. Toomaj et al. [14] investigated the Shannon differential entropy of the system’s lifetime, while [15] explored the Tsallis entropy of the same. Mesfioui et al. [16] also investigated the Tsallis entropy of coherent systems with identical properties, making this a fascinating area of current research. This research aims to investigate the uncertainty properties of mixed system lifetimes, specifically in terms of CRE. In contrast to Kayid and Alshehri’s prior work [15], our research centers on mixed systems composed of *n* components, all of which are operational at a given time *t*. Through the application of the system signature, we determine the CRE of a mixed system’s residual lifetime and establish an equation for the CRE of the conditional residual lifetime of the mixed system.

This paper presents the findings in the following structure: In Section 2, we introduce an expression for the CRE of a mixed system’s lifetime, assuming that all components of the system have survived up to time *t*. To achieve this, we utilize the powerful concept of system signature, which is particularly effective when component lifetimes are independent and identically distributed in a mixed system. Section 3 presents a series of useful bounds that further illuminate the properties of mixed systems. In Section 4, we propose a novel criterion for selecting the most suitable mixed system. Finally, we conclude with some closing reflections in Section 5.

## 2. CRE of the Residual Lifetime

This section introduces a groundbreaking concept known as the system signature, which we apply to define the CRE of a mixed system’s residual lifetime. A mixed system represents a stochastic blend of coherent systems, where a coherent system is a system if it does not have any irrelevant components and its structure function is monotone. In reliability engineering, the structure function is a mathematical function that describes the relationship between the reliability of a system and the reliability of its individual components. It is used to model complex systems and to evaluate their reliability. The signature of such a system is represented by an *n*-dimensional vector $p=(p_{1},\ldots ,p_{n})$, where $p_{i}=P\left(T=X_{i:n}\right),\;i=1,2,\ldots ,n,$ is the probability that the *i*-th failure causes the system failure. Here, $X_{i:n}$ denotes the lifetime of an *i*-out-of-*n* system, where the system fails when the *i*-th component failure occurs. Notice that $p_{1},\ldots ,p_{n}$ are non-negative real numbers that do not depend on the common cumulative distribution function (cdf) *F* and such that $\sum_{i=1}^{n}p_{i}=1.$

Suppose we examine a mixed system consisting of a set of independently and identically distributed (i.i.d.) component lifetimes $X_{1},\ldots ,X_{n}$, alongside a signature vector $p=(p_{1},\ldots ,p_{n})$ that is known in advance. Let $T_{t}^{n}=\left[T-t|X_{1:n}>t\right]$ denote the residual lifetime of the system under the condition that all components are operational at time *t*. Here, $X_{1:n}$ denotes the lifetime of the series system. By leveraging the results of [17], the survival function of $T_{t}^{n}$ can be elegantly expressed as follows:

(4) $$S_{T_{t}^{n}}\left(x\right)=\sum_{i=1}^{n}p_{i}S_{T_{t}^{i,n}}\left(x\right),\;x,t>0.$$

Here, the function $S_{T_{t}^{i,n}}\left(x\right)$ corresponds to the survival function of the residual lifetime of an *i*-out-of-*n* system, where $T_{t}^{i,n}=\left[X_{i:n}-t|X_{1:n}>t\right]$ denotes the time remaining for the *i*-th component to fail, given that all *n* components are operational at time *t*. The survival function of $T_{t}^{i,n}$ can be represented by the following expression:

STti,n(x)=∑k=0i−1nk1−St(x)kSt(x)n−k,x,t>0.

Our attention now shifts to investigating the CRE of the random variable $T_{t}^{n}$. To facilitate our analysis, we utilize the probability integral transformation $V=S_{t}\left(T_{t}^{n}\right),$ which recreates a vital part of our research. It is important to note that the transformation $U_{i:n}=S_{t}\left(T_{t}^{i,n}\right)$ follows a beta distribution with parameters n−i+1 and *i*. Moreover, its distribution function can be expressed as

(5) Gi(u)=∑k=0i−1ni(1−u)kun−k,0<u<1,i=1,…,n.

The upcoming theorem presents a concise expression for the CRE of $T_{t}^{n}$, utilizing the probability integral transformation and the beta distribution.

**Theorem** **1.** *We can express the CRE of $T_{t}^{n}$ as follows*

(6) $$E\left(T_{t}^{n}\right)=\int_{0}^{1}\frac{\psi (G_{V}\left(u\right))}{f_{t}\left(S_{t}^{-1}\left(u\right)\right)}du,\;t>0,$$

*where ψ(u)=−ulogu,0<u<1, and*

(7) $$G_{V}\left(u\right)=\sum_{i=1}^{n}p_{i}G_{i}\left(u\right),\;0\leq u\leq 1,$$

*represents the distribution function of $V=S_{t}\left(T_{t}^{n}\right).$ Here, V is the lifetime of the system with i.i.d. uniform distribution.*

**Proof.**  Using the change of variables $u=S_{t}\left(x\right)$, we can rewrite (1) and (4) as follows:

$$\begin{array}{ccc} E(T_{t}^{n}) & = & -\int_{0}^{\infty }S_{T_{t}^{n}}\left(x\right)\log S_{T_{t}^{n}}\left(x\right)dx\\ & = & -\int_{0}^{\infty }\left(\sum_{i=1}^{n}p_{i}S_{T_{t}^{i,n}}\left(x\right)\right)\log\left(\sum_{i=1}^{n}p_{i}S_{T_{t}^{i,n}}\left(x\right)\right)dx\\ & = & -\int_{0}^{1}\frac{\left(\sum_{i=1}^{n}p_{i}G_{i}\left(u\right)\right)\log\left(\sum_{i=1}^{n}p_{i}G_{i}\left(u\right)\right)}{f_{t}\left(S_{t}^{-1}\left(u\right)\right)}du, \end{array}$$

where $G_{i}\left(u\right)$ is the distribution function of $U_{i:n}=S_{t}\left(T_{t}^{i,n}\right)$ given in (5). Upon using Equation (7), we obtain the relation (6), which completes the proof. □

If $p=(0,\ldots ,0,1_{i},0,\ldots ,0),\;i=1,2,\ldots ,n,$ we arrive at a particular instance of Equation (6), which can be simplified to:

(8) $$E\left(T_{t}^{i,n}\right)=\int_{0}^{1}\frac{\psi (G_{i}\left(u\right))}{f_{t}\left(S_{t}^{-1}\left(u\right)\right)}du,\;t>0.$$

The next theorem is a straightforward consequence of Theorem 1 and is formulated in terms of the aging characteristics of the system’s components. It is worth noting that a random variable *X* is said to have an increasing failure rate (IFR) (decreasing failure rate (DFR)) if its hazard rate function λ(x)=f(x)/S(x) increases (decreases) in x>0.

**Theorem** **2.** *If X is IFR (DFR), then $E({T_{t}}^{1,n})$ is decreasing (increasing) in t.*

**Proof.**  From the definition of the hazard function, we have $f_{t}\left(S_{t}^{-1}\left(u\right)\right)=u\lambda_{t}\left(S_{t}^{-1}\left(u\right)\right)$ for 0<u<1. By substituting this expression into (6), we derive the subsequent alternative formulation for the CRE of $T_{t}^{1,n}$.

(9) $$\begin{array}{c} E\left({T_{t}}^{1,n}\right)=\int_{0}^{1}\frac{\psi (G_{V}\left(u\right))}{u\lambda_{t}\left(S_{t}^{-1}\left(u\right)\right)}du, \end{array}$$

for all t>0. One can show that $\lambda_{t}\left(S_{t}^{-1}\left(u\right)\right)=\lambda \left(S^{-1}\left(uS\left(t\right)\right)\right)$ for 0<u<1. If $t_{1}\leq t_{2}$, then $S^{-1}\left(uS\left(t_{1}\right)\right)\leq S^{-1}\left(uS\left(t_{2}\right)\right).$ Consequently, in the case where X is IFR (DFR), we can observe that

$$\begin{array}{ccc} \int_{0}^{1}\frac{\psi (G_{V}\left(u\right))}{u\lambda_{t_{1}}\left(S_{t_{1}}^{-1}\left(u\right)\right)}du & = & \int_{0}^{1}\frac{\psi (G_{V}\left(u\right))}{u\lambda (S^{-1}\left(uS\left(t_{1}\right)\right)}du\\ & \geq (\leq ) & \int_{0}^{1}\frac{\psi (G_{V}\left(u\right))}{u\lambda (S^{-1}\left(uS\left(t_{2}\right)\right)}dudu\\ & = & \int_{0}^{1}\frac{\psi (G_{V}\left(u\right))}{u\lambda_{t_{2}}\left(S_{t_{2}}^{-1}\left(u\right)\right)}du. \end{array}$$

Using (9), we conclude that $E\left({T_{t_{1}}}^{1,n}\right)\geq \left(\leq \right)E\left({T_{t_{2}}}^{1,n}\right)$ for all $t_{1}\leq t_{2}.$ This concludes the proof. □

The subsequent example illustrates how Theorems 1 and 2 can be implemented in practical applications to evaluate the CRE of a coherent system at the system level and investigate the system’s aging characteristics.

**Figure 1:**
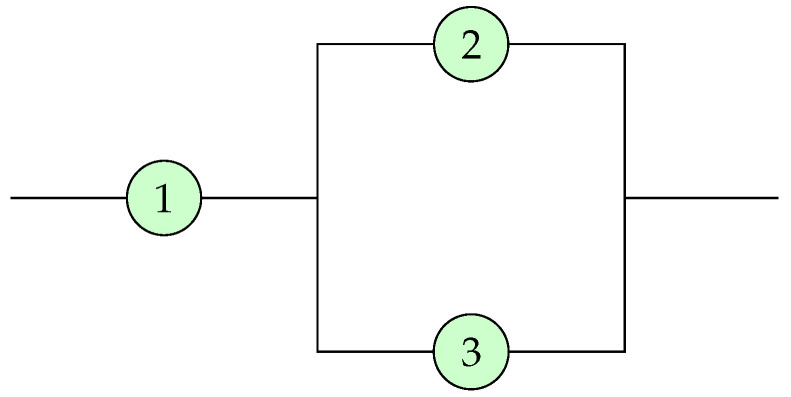
A system characterized by the system signature p=(1/3,2/3,0).

**Example** **1.** *Let p=(1/3,2/3,0) be the system signature of a coherent system as illustrated in Figure 1.*

*We suppose that the system’s component lifetimes are characterized by the survival function given by*

(10) $$S\left(t\right)=e^{-t^{k}},\;k,t>0.$$

*Upon performing some manipulation, we can represent the cumulative residual entropy of $T_{t}^{3}$ as*

$$E\left(T_{t}^{3}\right)=\int_{0}^{1}\frac{\psi (G_{V}\left(u\right))}{ku\left(t^{k}-\log u\right)}du,$$

**Figure 2:**
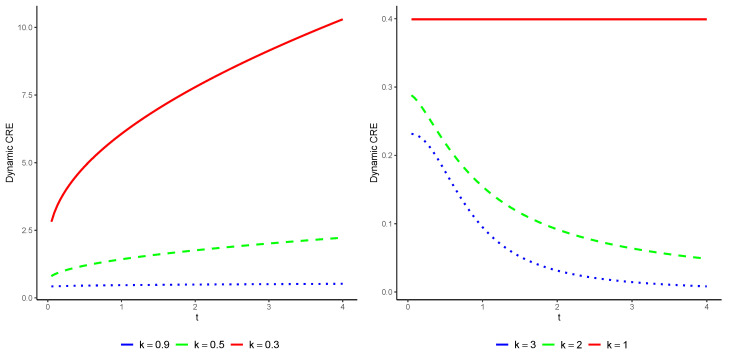
The exact values of $E(T_{t}^{3})$ with respect to *t* as illustrated in Example 1 for various values of k>0.

*for t>0. Regrettably, there is no explicit expression available for this relationship, and numerical methods must be employed to evaluate it. In Figure 2, we illustrate the CRE of ${T_{t}}^{1,3}$ as a function of time t for various values of k. It is well known that X has a decreasing failure rate (DFR) when 0<k<1 and an increasing failure rate (IFR) when k>1. As predicted by Theorem 2, we observe that $E(T_{t}^{3})$ increases with t for 0<k<1 and decreases for k>1.*

The following theorem demonstrates that the cumulative residual entropy of a mixed system lifetime, given that all components of the system are operational at time *t*, is dominated by the CRE of a new system lifetime.

**Theorem** **3.** *Let us examine a mixed system comprising i.i.d. component lifetimes that follow the IFR(DFR) distribution. Then, for all t>0, we have $E\left({T_{t}}^{1,n}\right)\leq \left(\geq \right)E\left(T\right)$.*

**Proof.**  Since X follows the IFR(DFR) distribution, we can utilize Theorem 3.B.25 of [18] to infer that $X\geq {\left(\leq \right)}_{d}X_{t}$, which further implies that

$$f_{t}\left(S_{t}^{-1}\left(u\right)\right)\geq \left(\leq \right)f\left(S^{-1}\left(u\right)\right),\;0<u<1,$$

for all t>0. Therefore, we have

(11) $$\int_{0}^{1}\frac{\psi (G_{V}\left(u\right))}{f_{t}\left(S_{t}^{-1}\left(u\right)\right)}du\leq \left(\geq \right)\int_{0}^{1}\frac{\psi (G_{V}\left(u\right))}{f(S^{-1}\left(u\right))}du,\;t>0,$$

since $\psi (G_{V}\left(u\right))\geq 0$ for all 0<u<1. Using (6) and (11), we conclude that $E\left(T_{t}^{n}\right)\leq \left(\geq \right)E\left(T\right)$, which completes the proof. □

In engineering reliability, the concept of duality proves to be quite useful for reducing the computational burden of calculating the signatures of all coherent systems of a particular size by approximately half. If the minimal path sets of a mixed system *A* represent the minimal cut sets of another mixed system *B*, and vice versa, then we refer to mixed system *A* as the dual of mixed system B. Specifically, if a mixed system has a lifetime of $T_{t}^{n}$ with a signature of $p=(p_{1},\ldots ,p_{n})$, then the signature of its dual system with a lifetime of ${T_{t}}^{D,n}$ is $p^{D}=\left(p_{n},\ldots ,p_{1}\right),$ (see, Samaniego [19]). Leveraging the concept of duality, the upcoming theorem reduces the computational complexity involved in calculating the residual CRE of mixed systems.

**Theorem** **4.** *If the condition $f_{t}\left(S_{t}^{-1}\left(v\right)\right)=f_{t}\left(S_{t}^{-1}\left(1-v\right)\right)$ holds for all 0<v<1 and t, we can deduce that $E\left(T_{t}^{n}\right)=E\left({T_{t}}^{D,n}\right)$ for all p and n.*

**Proof.**  It is worth noting that $G_{i}\left(1-v\right)=G_{n-i+1}\left(v\right)$ holds true for all i=1,…,n and 0<v<1. By assuming that $f_{t}\left(S_{t}^{-1}\left(v\right)\right)=f_{t}\left(S_{t}^{-1}\left(1-v\right)\right)$ for all 0<v<1, we can use (6) to obtain

$$\begin{array}{ccc} E({T_{t}}^{D,n}) & = & -\int_{0}^{1}\frac{\left(\sum_{i=1}^{n}p_{n-i+1}G_{i}\left(u\right)\right)\log\left(\sum_{i=1}^{n}p_{n-i+1}G_{i}\left(u\right)\right)}{f_{t}\left(S_{t}^{-1}\left(u\right)\right)}du\\ & = & -\int_{0}^{1}\frac{\left(\sum_{r=1}^{n}p_{r}G_{n-r+1}\left(u\right)\right)\log\left(\sum_{r=1}^{n}p_{r}G_{n-r+1}\left(u\right)\right)}{f_{t}\left(S_{t}^{-1}\left(u\right)\right)}du\\ & = & -\int_{0}^{1}\frac{\left(\sum_{r=1}^{n}p_{r}G_{r}\left(1-u\right)\right)\log\left(\sum_{r=1}^{n}p_{r}G_{r}\left(1-u\right)\right)}{f_{t}\left(S_{t}^{-1}\left(1-u\right)\right)}du\\ & = & -\int_{0}^{1}\frac{\left(\sum_{r=1}^{n}p_{r}G_{r}\left(v\right)\right)\log\left(\sum_{r=1}^{n}p_{r}G_{r}\left(v\right)\right)}{f_{t}\left(S_{t}^{-1}\left(v\right)\right)}dv\\ & = & E(T_{t}^{n}). \end{array}$$

This completes the proof. □

Hereafter, we explore the partial ordering of the conditional lifetimes of two mixed systems, taking into account their uncertainties. We investigate the CRE ordering of the residual lifetimes of the two systems based on various existing orderings between the lifetimes of the components and their signature vectors. The following theorem compares the CREs of residual lifetimes of two mixed systems. Let *X* and *Y* be two non-negative random variables with distribution functions *F* and G, respectively. Let $F^{-1}$ and $G^{-1}$ be the right continuous inverses of *F* and G, respectively. We recall that *X* is smaller than *Y* in the dispersive order (denoted by $X\leq_{d}Y$) if $F^{-1}\left(u\right)-F^{-1}\left(v\right)\leq G^{-1}\left(u\right)-G^{-1}\left(v\right),\;0<v\leq u<1.$

**Theorem** **5.** *Let us consider two mixed systems with the same signatures and n i.i.d. component lifetimes $X_{1},\ldots ,X_{n}$ and $Y_{1},\ldots ,Y_{n}$. The residual lifetimes of these systems are denoted as $T_{t}^{X,n}=\left[T-t|X_{1:n}>t\right]$ and $T_{t}^{Y,n}=\left[T-t|Y_{1:n}>t\right]$, respectively. If $X\leq_{d}Y$ and either X or Y is IFR, then we can conclude that $E\left(T_{t}^{X,n}\right)\leq E\left(T_{t}^{Y,n}\right)$ for all t.*

**Proof.**  Using the relation (6), we can show that $X_{t}\leq_{d}Y_{t}$, which is sufficient to establish the desired inequality between the cumulative residual entropies. Since we assume that either *X* or *Y* is IFR and $X\leq_{d}Y$, Theorem 5 of Ebrahimi and Kirmani [20] implies that $X_{t}\leq_{d}Y_{t}.$ Thus, the proof is complete. □

The following example showcases an application of Theorem 5.

**Figure 3:**
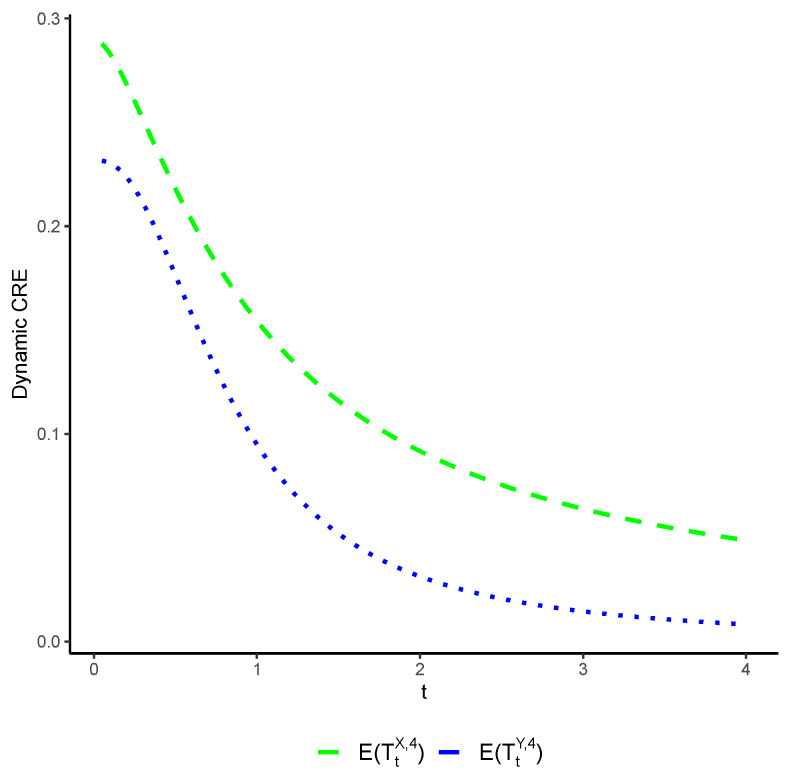
The exact values of $E(T_{t}^{X,4})$ (blue color) and $E(T_{t}^{Y,4})$ (green color) with respect to t.

**Example** **2.** *Consider two coherent systems characterized by their residual lifetimes, denoted by $T_{t}^{X,4}$ and $T_{t}^{Y,4}$, respectively, which share the common signature $p=(\frac{1}{2},\frac{1}{4},\frac{1}{4},0)$. Let X and Y follow the Weibull distributions with shape and scale parameters (3,1) and (2,1), respectively. It is noteworthy that $X\leq_{d}Y$ and both distributions belong to the class of increasing failure rate distributions. Consequently, invoking Theorem 5 leads to the conclusion that $E\left(T_{t}^{X,4}\right)\leq E\left(T_{t}^{Y,4}\right)$ for all t>0. The associated dynamic CRE measure for these systems are depicted in Figure 3.*

## 3. Bounds for CRE of the Residual Lifetime

When dealing with highly complex systems with a large number of components, computing the cumulative residual entropy $E(T_{t}^{n})$ of a mixed system can be challenging. This is a common practical issue that arises in many applications. To address this challenge, researchers have recently developed bounds for the uncertainty of the lifetimes of mixed systems, as discussed in studies such as [14], and their related references. In the following theorem, we provide bounds for the residual cumulative residual entropy of a mixed system’s lifetime, in terms of the residual entropy of the parent distribution $E(X_{t})$. These bounds can be valuable for approximating the lifetime of a mixed system, particularly in scenarios where the computation of its exact cumulative residual entropy is challenging.

**Proposition** **1.** *Consider a mixed system with the same signature $p=(p_{1},\ldots ,p_{n})$ and n i.i.d component lifetimes $X_{1},\ldots ,X_{n}$ with residual lifetime denoted as $T_{t}^{n}=\left[T-t|X_{1:n}>t\right].$ Then,*

$$\begin{array}{c} B_{1}E\left(X_{t}\right)\leq E\left(T_{t}^{n}\right)\leq B_{2}E\left(X_{t}\right) \end{array}$$

*where $B_{1}=\inf_{u\in (0,1)}\frac{\psi (G_{V}\left(u\right))}{\psi (u)}$, $B_{2}=\sup_{u\in (0,1)}\frac{\psi (G_{V}\left(u\right))}{\psi (u)}$ and ψ(u)=−ulog(u).*

**Proof.**  We can obtain an upper bound for $E(T_{t}^{n})$ of a mixed system with *n* i.i.d. component lifetimes by using (6). Specifically, we have

$$\begin{array}{ccc} E(T_{t}^{n}) & = & \int_{0}^{1}\frac{\psi (G_{V}\left(u\right))}{f_{t}\left(S_{t}^{-1}\left(u\right)\right)}du\\ & = & \int_{0}^{1}\frac{\psi (G_{V}\left(u\right))}{\psi (u)}\frac{\psi (u)}{f_{t}\left(S_{t}^{-1}\left(u\right)\right)}du\\ & \leq & \underset{u\in (0,1)}{\sup}\frac{\psi (G_{V}\left(u\right))}{\psi (u)}\int_{0}^{1}\frac{\psi (u)}{f_{t}\left(S_{t}^{-1}\left(u\right)\right)}du\\ & = & B_{2}E\left(X_{t}\right), \end{array}$$

where $B_{2}$ is a constant that depends on the distribution of *V*. Similarly, we can obtain a lower bound for $E(T_{t}^{n})$ using the same approach. □

In the above theorem, the lower bound $B_{1}$ is determined by the minimum value of the ratio $\psi (G_{V}\left(u\right))/\psi (u)$, evaluated at u∈(0,1). The upper bound $B_{2}$ is determined by the maximum value of the same ratio, evaluated at u∈(0,1). These bounds provide a useful tool for estimating the residual cumulative residual entropy of a mixed system based on the cumulative residual entropy of its component lifetimes.

**Remark** **1.** *We would like to emphasize that the lower bound $B_{1}$ in Proposition 1 is equal to zero for all the mixed systems with i.i.d. components and signature $\left(p_{1},\ldots ,p_{n}\right)$ satisfying $s_{1}=0$ or $s_{n}=0$. In particular, it is zero for all the coherent systems with n>1 i.i.d. components; (see [8]).*

Here, we derive a comprehensive lower bound for the CRE of $T_{t}^{n}$ by leveraging the system signature and CRE of *k*-out-of-*n* systems.

**Proposition** **2.** *Suppose we have a mixed system with a signature of $\left(p_{1},\ldots ,p_{n}\right)$ composed of n i.i.d. components, denoted by $T_{t}^{n}$. Then, we can make the following statement:*

(12) $$E\left(T_{t}^{n}\right)\geq E_{L}\left(T_{t}^{n}\right),$$

*where $E_{L}\left(T_{t}^{n}\right)=\sum_{i=1}^{n}p_{i}E\left(T_{t}^{i,n}\right).$*

**Proof.**  Using Samaniego’s representation, we can express the distortion function $G_{V}\left(v\right)$ associated with the signature vector $p=(p_{1},\ldots ,p_{n})$ and the component lifetimes $X_{1},X_{2},\ldots ,X_{n}$ as $G_{V}\left(v\right)=\sum_{i=1}^{n}p_{i}G_{i:n}\left(v\right)$, where $G_{i:n}\left(v\right)$ is the distortion function associated with the *i*th order statistic $X_{i:n}$. By applying (6) and the concavity of the distortion function ψ(u)=−ulog(u), we obtain the lower bound:

$$\begin{array}{cc} E(T_{t}^{n}) & =\int_{0}^{1}\frac{\psi (G_{V}\left(u\right))}{f_{t}\left(S_{t}^{-1}\left(u\right)\right)}du\\ \; & \geq \int_{0}^{1}\frac{\sum_{i=1}^{n}p_{i}\psi \left(G_{i:n}\left(u\right)\right)}{f_{t}\left(S_{t}^{-1}\left(u\right)\right)}du\\ \; & =\sum_{i=1}^{n}p_{i}E\left(T_{t}^{i,n}\right), \end{array}$$

where $E(T_{t}^{i,n})$ is the cumulative residual entropy of $T_{t}^{i,n}$. □

This bound shows that the cumulative residual entropy of the mixed system is lower-bounded by a linear combination of the cumulative residual entropies of the component lifetimes, with weights given by the signature vector. This result can be particularly useful when the signature vector is known and the component lifetimes have a monotone hazard rate function, as it allows for a direct estimation of the cumulative residual entropy of the mixed system without the need for complex computations. It is worth noting that equality in (12) is valid for *i*-out-of-*n* systems, where we have $p_{j}=0$ for j≠i, $p_{j}=1$ for j=i, and $E\left(T_{t}^{n}\right)=E\left(T_{t}^{i,n}\right)$. When the lower bounds in both parts of Theorems 1 and 2 can be computed, one may use the maximum of the two lower bounds.

**Example** **3.** *Let us consider a coherent system with the signature $p=(0,\frac{3}{10},\frac{5}{10},\frac{2}{10},0)$, comprising n=5 i.i.d. component lifetimes drawn from a uniform distribution in the interval [0,1]. Let $T_{t}^{5}$ denote the residual lifetime of this system, defined as $T_{t}^{5}=\left[T-t|X_{1:5}>t\right]$. Remarkably, due to Remark 1, we have $B_{1}=0$, while $B_{2}=18.21$ and $E\left(X_{t}\right)=\frac{1}{4}\left(1-t\right),\;0<t<1.$ Utilizing Theorem 1, we conclude that the conditional residual entropy of $T_{t}^{1,5}$ is bounded as follows:*

(13) $$0\leq E(T_{t}^{1,5})\leq 4.55\left(1-t\right),\;0<t<1.$$

*In addition, since $\sum_{i=1}^{n}p_{i}E\left(U_{i:n}\right)=0.16,$ the lower bound given in (12) can be expressed as:*

(14) $$E\left(T_{t}^{1,5}\right)\geq 0.16\left(1-t\right),$$

*for all 0<t<1. By combining the lower bound in (14) and the upper bound in (13), we obtain the following inequality for the conditional CRE of $T_{t}^{1,5}$:*

$$0.16\left(1-t\right)\leq E\left(T_{t}^{1,5}\right)\leq 4.55\left(1-t\right),$$

*for all 0<t<1. This provides a tight bound on the conditional CRE of $T_{t}^{1,5}$ for all values of t in the interval (0,1).*

## 4. Preferable System

In pairwise comparisons, the typical stochastic ordering may not suffice due to the intrinsic nature of certain system structures. Several pairs of systems remain incomparable under any of the conventional stochastic indices. To overcome this limitation, we delve into various metrics for comparing the performance of systems.

In the next section, we present a novel approach for comparing information measures. When comparing systems, engineers generally prefer those systems with longer operational times.

Therefore, it is essential to ensure that the competing systems have similar characteristics. Furthermore, assuming identical characteristics, a parallel system design is the most suitable option as it provides superior performance and a longer residual lifetime compared to other systems. Concerning reliability, we can utilize (4) to establish the following property:

$$\begin{array}{c} P\left(T_{t}^{1,n}>x\right)\leq P\left(T_{t}^{n}>x\right)\leq P\left(T_{t}^{n,n}>x\right),\;x>0, \end{array}$$

for all t>0. Rather than relying on pairwise comparisons, we can instead search for a system whose structure or distribution is more akin to that of the parallel system. In essence, we seek to answer the following question: which of these systems bears greater similarity (or proximity) to the parallel system’s configuration while being dissimilar to the series system’s configuration? To address this query, we utilize the concept of relative CRE discrimination. To simplify our analysis, we use the distance measure between two distributions proposed by Toomaj et al. [8].

**Definition** **1.** *Suppose that $X_{t}=\left[X-t|X>t\right]$ and $Y_{t}=\left[Y-t|Y>t\right]$ are two non-negative residual random variables that share a common support, with CDFs $F_{t}$ and $G_{t}$, respectively. In such a scenario, the Symmetric Kullback–Leibler (SKL) divergence is defined as follows:*

(15) $$\begin{array}{ccc} \mathrm{SCE}(X_{t},Y_{t}) & = & \int_{0}^{\infty }\left[{\bar{F}}_{t}\left(x\right)-{\bar{G}}_{t}\left(x\right)\right]\log\frac{{\bar{F}}_{t}\left(x\right)}{{\bar{G}}_{t}\left(x\right)}dx. \end{array}$$

The metric (15) that we have introduced is both non-negative and symmetric and satisfies the condition $\mathrm{SCE}(X_{t},Y_{t})=0$ if and only if ${\bar{F}}_{t}\left(x\right)={\bar{G}}_{t}\left(x\right)$ almost everywhere. Based on this, we propose the following Symmetric Dynamic Distance Measure (DDSM) for $T_{t}^{n}$:

(16) $$\mathrm{DDSM}\left(T_{t}^{n}\right)=\frac{\mathrm{SCE}\left(T_{t}^{n},T_{t}^{1,n}\right)-\mathrm{SCE}\left(T_{t}^{n},T_{t}^{n,n}\right)}{\mathrm{SCE}(T_{t}^{1,n},T_{t}^{n,n})}.$$

Proposition 3 establishes that $-1\leq \mathrm{DS}(T_{t}^{n})\leq 1$. It follows that $\mathrm{DS}(T_{t}^{n})=1$ if and only if $T_{t}^{n}{=}_{st}T_{t}^{n,n}$, and $\mathrm{DS}(T_{t}^{n})=-1$ if and only if $T_{t}^{n}{=}_{st}T_{t}^{1,n}$. In simpler terms, we can infer that a value of $\mathrm{DS}(T_{t}^{n})$ closer to 1 indicates that the distribution of $T_{t}^{n}$ is more similar to that of a parallel system. Conversely, a value of $\mathrm{DS}(T_{t}^{n})$ closer to −1 suggests that the distribution of $T_{t}^{n}$ is more similar to that of a series system. With this understanding, we propose the following definition. For the sake of simplicity, we will henceforth examine two mixed systems, each comprised of *n* i.i.d. component lifetimes and possessing signatures $p_{1}$ and $p_{2}$. We denote the residual lifetimes of these systems as $T_{1,t}^{n}$ and $T_{2,t}^{n}$, respectively.

**Definition** **2.** *We say that $T_{2,t}^{n}$ is more preferable than $T_{1,t}^{n}$ in terms of the Dynamic Distance Symmetric Measure, denoted by $T_{1,t}^{n}\leq_{DDSM}T_{2,t}^{n}$, at time t, denoted by $T_{1,t}^{n}\leq_{DDSM}T_{2,t}^{n}$, if and only if $\mathrm{DDSM}\left(T_{1,t}^{n}\right)\leq \mathrm{DDSM}\left(T_{2,t}^{n}\right)$ for all t>0. more preferable than $T_{1,t}^{n}$ in the Distance Symmetric Measure, denoted by $T_{1,t}^{n}\leq_{DDSM}T_{2,t}^{n}$, if $\mathrm{DDSM}\left(T_{1,t}^{n}\right)\leq \mathrm{DDSM}\left(T_{2,t}^{n}\right)$.*

It is worth noting that $\mathrm{DDSM}\left(T_{1,t}^{n}\right)=\mathrm{DDSM}\left(T_{2,t}^{n}\right)$ does not necessarily imply that $T_{1,t}^{n}{=}_{st}T_{2,t}^{n}$. Under the conditions stipulated in Definition 2, we define $\mathrm{DDS}\left(T_{t}^{n}\right)=\mathrm{SCE}\left(T_{t}^{n},T_{t}^{1,n}\right)-\mathrm{SCE}\left(T_{t}^{n},T_{t}^{n,n}\right)$. In the case of i.i.d. components, Equation (15) and the aforementioned transformations result in the following expression:

(17) $$\begin{array}{ccc} \mathrm{SCE}(T_{t}^{n},T_{t}^{i,n}) & = & \int_{0}^{1}\frac{\left[\psi \left(G_{V}\left(u\right)\right)-\psi \left(G_{i:n}\left(u\right)\right)\right]}{f_{t}\left(S_{t}^{-1}\left(u\right)\right)}\log\frac{\psi (G_{V}\left(u\right))}{\psi (G_{i:n}\left(u\right))}du, \end{array}$$

for i=1,n. Then, from (17), we obtain

$$\begin{array}{ccc} \mathrm{DDS}(T_{t}^{n}) & = & \int_{0}^{1}\frac{\left[\psi \left(G_{V}\left(u\right)\right)-\psi \left(G_{1:n}\left(u\right)\right)\right]}{f_{t}\left(S_{t}^{-1}\left(u\right)\right)}\log\frac{\psi (G_{V}\left(u\right))}{\psi (G_{1:n}\left(u\right))}du\\ & - & \int_{0}^{1}\frac{\left[\psi \left(G_{V}\left(u\right)\right)-\psi \left(G_{n:n}\left(u\right)\right)\right]}{f_{t}\left(S_{t}^{-1}\left(u\right)\right)}\log\frac{\psi (G_{V}\left(u\right))}{\psi (G_{n:n}\left(u\right))}du, \end{array}$$

and

$$\mathrm{SCE}\left(T_{t}^{1,n},T_{t}^{n,n}\right)=\int_{0}^{1}\frac{\left[\psi \left(G_{n:n}\left(u\right)\right)-\psi \left(G_{1:n}\left(u\right)\right)\right]}{f_{t}\left(S_{t}^{-1}\left(u\right)\right)}\log\frac{\psi (G_{n:n}\left(u\right))}{\psi (G_{1:n}\left(u\right))}du.$$

In the case where the components are i.i.d., we have $\psi \left(G_{1:n}\left(u\right)\right)=v^{n}$ and $\psi \left(G_{n:n}\left(u\right)\right)=1-{\left(1-v\right)}^{n}$. Given that $T_{t}^{1,n}\leq_{st}T_{t}\leq_{st}T_{t}^{n,n}$ holds true for any system $T_{t}$, and by recalling Lemma 1 of Toomaj et al. [8], we can obtain the following fascinating outcome.

**Proposition** **3.** *It holds that $\mathrm{SCE}\left(T_{t}^{n},T_{t}^{i,n}\right)\leq \mathrm{SCE}\left(T_{t}^{1,n},T_{t}^{n,n}\right)$, for i=1,n.*

We immediately have the following theorem.

**Theorem** **6.** *Under the conditions of Definition 2, if the component lifetimes have a common exponential distribution, then the dynamic distance symmetric measure does not depend on time t, i.e., $\mathrm{DDSM}\left(T_{t}^{n}\right)=\mathrm{DDSM}\left(T^{n}\right),$ for all t>0.*

**Proof.**  Upon using the memoryless property of exponential distribution, we obtain $f_{t}\left(S_{t}^{-1}\left(u\right)\right)$ $=f(S^{-1}\left(u\right))$ for all t>0. Therefore, we have the result. □

**Example** **4.** *Suppose we have two systems with different signatures, denoted by $T_{1,t}^{4}$ and $T_{2,t}^{4}$, whose components’ lifetimes follow the standard exponential distribution with signatures $p_{1}=\left(0,2/3,1/3,0\right)$ and $p_{2}=\left(1/4,1/4,1/2,0\right)$, respectively. Despite being incomparable in the usual stochastic orders, we can evaluate their relative performance using the in terms of Dynamic Distance Symmetric Measure. By computing the values of $\mathrm{DDSM}(T_{1,t}^{4})$ and $\mathrm{DDSM}(T_{2,t}^{4})$, we find that the latter system outperforms the former with a higher score of −0.1505, compared to −0.1918 for the former. This suggests that the system with signature $p_{1}=\left(1/4,1/4,1/2,0\right)$ is a better choice than the one with signature $p_{2}=\left(0,2/3,1/3,0\right)$, as it is more similar to a parallel system.*

**Theorem** **7.** *If $p_{1}\leq_{st}p_{2}$, we can assert that $T_{1,t}^{n}\leq_{DDSM}T_{2,t}^{n}$.*

**Proof.**  Excitingly, the desired result can be derived from Theorem 2.3 of Khaledi and Shaked [17], as $p_{1}\leq_{st}p_{2}$ implies $T_{t}^{1,n}\leq_{st}T_{1,t}^{n}\leq_{st}T_{2,t}^{n}\leq_{st}T_{t}^{n,n}$. By applying Lemma 1 of Toomaj et al. [8], we obtain $\mathrm{SCE}\left(T_{1,t}^{n},T_{t}^{1,n}\right)\leq \mathrm{SCE}\left(T_{2,t}^{n},T_{t}^{1,n}\right)$ and $\mathrm{SCE}\left(T_{1,t}^{n},T_{t}^{n,n}\right)\geq \mathrm{SCE}\left(T_{2,t}^{n},T_{t}^{n,n}\right)$, leading to the desired result due to relation (16). □

An intriguing finding is that DDSM comparison can serve as a prerequisite for the conventional stochastic order, which enables us to compare systems that cannot be compared using the conventional stochastic order. In particular, if $T_{1,t}^{n}$ and $T_{2,t}^{n}$ are two coherent (or mixed) systems based on component lifetimes $X_{1},\ldots ,X_{n}$, and $T_{1,t}^{n}\leq_{st}T_{2,t}^{n}$, then we can conclude that $T_{1,t}^{n}\leq_{DDSM}T_{2,t}^{n}$. Hence, the DDSM order provides us with a means of comparing systems that would otherwise be challenging to compare. Remarkably, if $T_{1,t}^{n}{=}_{st}T_{2,t}^{n}$, then $T_{1,t}^{n}{=}_{DDSM}T_{2,t}^{n}$, highlighting the potential of DDSM comparison in system analysis.

## 5. Conclusions

Recent years have seen an increase in interest in measuring the uncertainty associated with engineering systems’ lifetimes. An assessment of predictability over a system’s lifetime can be determined through this criterion. The CRE measure, being an extension of the Shannon entropy, proves to be a highly appealing tool in such scenarios. In this paper, we introduced an approach for calculating the CRE of a system’s lifetime, with the assumption that all system components are functional at a given time *t*. Furthermore, we explored several properties of this metric, established various bounds, and examined the relationships between the residual lifetimes of two mixed systems in terms of their CRE uncertainties. Our research offers new insights into the field of reliability engineering and provides practical implications for the design and optimization of complex systems. To illustrate our findings, we provided several examples. Lastly, we introduced a criterion, based on relative CRE, to select a preferable system that bears a closer resemblance to the parallel system.

## Data Availability

No new data were created or analyzed in this study. Data sharing is not applicable to this article.
